# Identification of a Major QTL Controlling Maize Lateral Root Branching Number Through Integrated BSA-Seq and Transcriptome Analysis

**DOI:** 10.3390/ijms27156962

**Published:** 2026-08-03

**Authors:** Deyu Liu, Qihang Wang, Mengmeng Cao, Zihui Sun, Dafeng Peng, Lianghai Sun, Yongqiang Chen, Yafei Wang, Zhanyong Guo, Zhanhui Zhang, Jihua Tang

**Affiliations:** 1 State Key Laboratory of Wheat-Maize Double Cropping for High-Efficiency Production, College of Agronomy, Henan Agricultural University, Zhengzhou 450002, China; ldy1796@163.com (D.L.); wqh1162906881@163.com (Q.W.); 15036959429@163.com (M.C.); sunzh2026@126.com (Z.S.); 18586543924@163.com (D.P.); a2332660688@163.com (L.S.); chenyongqiang@henau.edu.cn (Y.C.); yafeiwang@henau.edu.cn (Y.W.); gzy@henau.edu.cn (Z.G.); 2 The Shennong Laboratory, Zhengzhou 450002, China

**Keywords:** maize (*Zea mays* L.), lateral root, QTL mapping, BSA-seq, transcriptome analysis

## Abstract

Maize lateral roots play essential roles in plant anchorage, mechanical support, and water and nutrient acquisition. However, the genetic mechanisms underlying lateral root development remain unclear. In this study, an F_2_ population was generated by crossing the inbred lines B111, which exhibits abundant lateral roots on brace roots, and T4691, which lacks lateral roots on brace roots, to map loci controlling lateral root development. Phenotypic analysis of the F_2_ population revealed that lateral root branching number (LRN) exhibited quantitative variation. Bulked segregant analysis sequencing (BSA-seq) identified two major QTLs for LRN on chromosomes 7 and 9. Of them, the QTL on chromosome 9, *qLRN9*, showed stronger significance. Through molecular marker analysis, *qLRN9* was further narrowed down to an approximately 1.63 Mb marker interval. To identify the candidate genes, transcriptome analysis was performed using brace root samples with lateral root branches collected from the B111 inbred line at three lateral root developmental stages. In the ~1.63-marker interval, *Zm00001eb379100*, *Zm00001eb378700*, *Zm00001eb379070* and *GRFTF13* were screened for possible candidate genes by integrated analysis of QTL mapping and transcriptome data. These findings provide valuable genetic resources and molecular markers for improving maize root architecture, lodging resistance, and drought tolerance.

## 1. Introduction

Maize (*Zea mays* L.) is one of the world’s three major cereal crops, with an annual global production exceeding one billion tons. It provides approximately 15% of protein and 20% of caloric intake for humans. In addition, maize serves as an important source of animal feed and industrial raw materials [1]. In China, maize ranks first among cereal crops in both planting area and total production, playing a crucial role in ensuring national food security [2]. However, the growth and productivity of maize are severely constrained by various abiotic stresses, among which lodging and drought are the two most significant limiting factors [3,4]. Lodging can cause 15% to 30% yield loss, accompanied by grain mildew, quality degradation and increased harvesting costs [5,6,7]. Drought stress inhibits plant growth and development, causes leaf dehydration and wilting, reduces plant height and leaf area, decreases material accumulation, and, under severe conditions, may even result in plant death [3,8]. The effects of drought on maize productivity vary depending on its severity, duration, and developmental stage at which it occurs [9]. Because maize plants have a high center of gravity and large ears that increase mechanical load, their structural stability depends heavily on the anchorage provided by the root system [10]. With the continued increase in planting density and the growing frequency of extreme weather events, improving lodging resistance and drought tolerance has become a major objective in modern maize breeding.

The maize root system consists of primary, seminal, lateral and brace roots [11]. Brace and crown roots originate from the stem nodes, grow downward into the soil, and subsequently produce lateral roots, together forming a robust supporting structure [12]. The root system is the primary organ responsible for anchoring the plant and maintaining mechanical stability [13]. Improvements in root system architecture have played a significant role in increasing planting density and grain yield in modern maize breeding programs [14]. Beyond providing mechanical support, root architecture—particularly lateral root development—determines the overall size and spatial distribution of the root system, thereby influencing the contact area between roots and the soil and ultimately affecting water and nutrient uptake. Lateral roots originate from the pericycle cells of the primary root or nodal roots and develop through a series of developmental stages, including primordium initiation, emergence through the overlying tissues, and subsequent elongation, ultimately forming an extensive absorptive root network [15]. The density and length of lateral roots are closely associated with the plant’s capacity to acquire water and nutrients. Under conditions of heterogeneous soil nutrient distribution or water deficit, a well-developed lateral root system enhances the acquisition of deep soil water and relatively immobile phosphorus [16]. Consistent with this, increased lateral root length has been shown to improve the drought tolerance of maize [17].

To date, studies investigating the molecular mechanisms underlying root development have primarily focused on the model plant *Arabidopsis* and on the primary and seminal roots of maize. In contrast, the genetic regulatory mechanisms governing lateral root initiation remain poorly understood. Insights into lateral root development have largely been derived from *Arabidopsis*, and many of the underlying regulatory pathways are conserved in cereal crops, including maize. Auxin is the central phytohormone controlling lateral root initiation. The initiation of lateral root primordia requires the establishment of a local auxin maximum within specific pericycle cells, which is achieved through polar auxin transport mediated by PIN-FORMED (PIN) transport proteins [18]. Following nuclear perception of auxin by the receptor complex TIR1/AFB, Aux/IAA transcriptional repressors are degraded via the ubiquitin–proteasome pathway, thereby releasing the AUXIN RESPONSE FACTOR (ARF) transcription factors to activate the expression of downstream auxin-responsive genes [19]. Together, ARF and Aux/IAA proteins constitute the core signaling module of the auxin–auxin response pathway [20]. In *Arabidopsis*, ARF7 and ARF19 activate members of the LATERAL ORGAN BOUNDARIES DOMAIN/ASYMMETRIC LEAVES2-LIKE (LBD/ASL) transcription factor family [21], which subsequently activates the expression of cell cycle-related genes to promote pericycle cell division and lateral root primordium formation [22].

In our previous study, we identified the maize inbred line B111, which exhibits a high density of lateral roots on both the underground root system and brace roots. This distinctive phenotype provides an excellent genetic resource for dissecting the molecular basis of lateral root branching. In this study, B111 (high lateral root branching density) was crossed with the inbred line T4691 (low lateral root branching density) to generate an F_2_ segregation population. Bulked segregant analysis sequencing (BSA-seq) combined with transcriptome analysis was subsequently employed to identify candidate genes regulating lateral root development. Our findings provide new insights into the genetic regulation of maize lateral root development and offer valuable genetic resources for improving root architecture, lodging resistance, and drought tolerance through molecular breeding.

## 2. Results

### 2.1. Phenotypic Analysis of Inbred Line B111 and the Segregating Population

**Figure 1:**
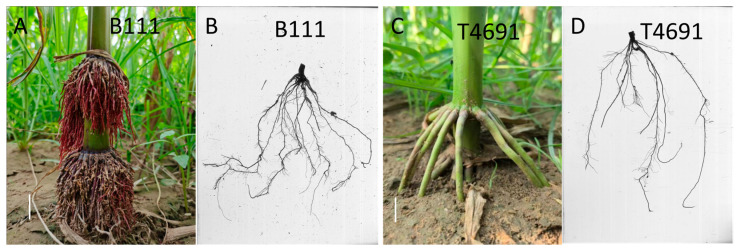
Phenotypic characteristics of lateral roots in maize inbred lines B111 and T4691: (**A**) Lateral root phenotypes on the brace root in inbred line B111. Bar = 3 cm. (**B**) Lateral root phenotypes of inbred line B111 at seedling stage. (**C**) Lateral root phenotypes on the brace root in inbred line T4691. Bar = 3 cm. (**D**) Lateral root phenotypes of inbred line T4691 at seedling stage.

In our previous study, we screened the maize inbred line B111, which exhibits a high density of lateral roots on its brace roots (Figure 1). Further phenotypic characterization revealed that B111 also develops significantly more lateral roots at the seedling stage than the inbred line T4691. An F_2_ segregating population was subsequently generated by crossing B111 and T4691. Analysis of this population showed that lateral root number exhibited a continuous, approximately normal distribution, suggesting that this trait is quantitatively inherited. To investigate the contribution of lateral root number to drought tolerance, four F_2:3_ lines with either high lateral root branching (MLR) or low lateral root branching (LLR) were selected and subjected to drought stress treatment at the seedling stage.

**Figure 2:**
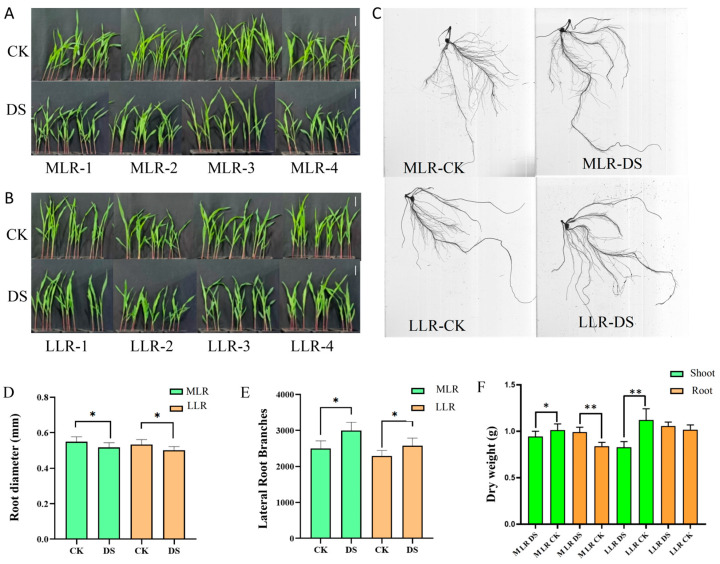
Drought stress treatments on the MLR and LLR F_2:3_ lines. (**A**) The effects of drought stress on the four MLR lines. Bar = 3 cm. (**B**) The effects of drought stress on the four LLR lines. Bar = 3 cm. (**C**) The effects of drought stress on lateral root characters of MLR and LLR lines. (**D**–**F**) The effects of drought stress on the lateral root diameter, lateral root branches, and shoot and root dry weight of MLR and LLR lines. * and ** indicate that the corresponding lateral root diameter, lateral root branches, and dry weight of the tested F_2:3_ lines under drought stress are significantly different from the control at *p* < 0.05 and *p* < 0.01 levels, respectively. CK and DS represent the control group and drought stress treatment group, respectively. Error bars represent standard deviation (SD).

Under well-watered conditions, no significant morphological differences were observed between the MLR and LLR (Figure 2). In contrast, drought stress induced distinct differences in root growth between the two groups. MLR lines exhibited a higher density of lateral roots and greater root dry weight under drought conditions. Compared with MLR lines, LLR lines displayed more severe leaf rolling and a greater reduction in shoot dry weight.

### 2.2. QTL Mapping by BSA-Seq and Molecular Marker Analysis

**Figure 3:**
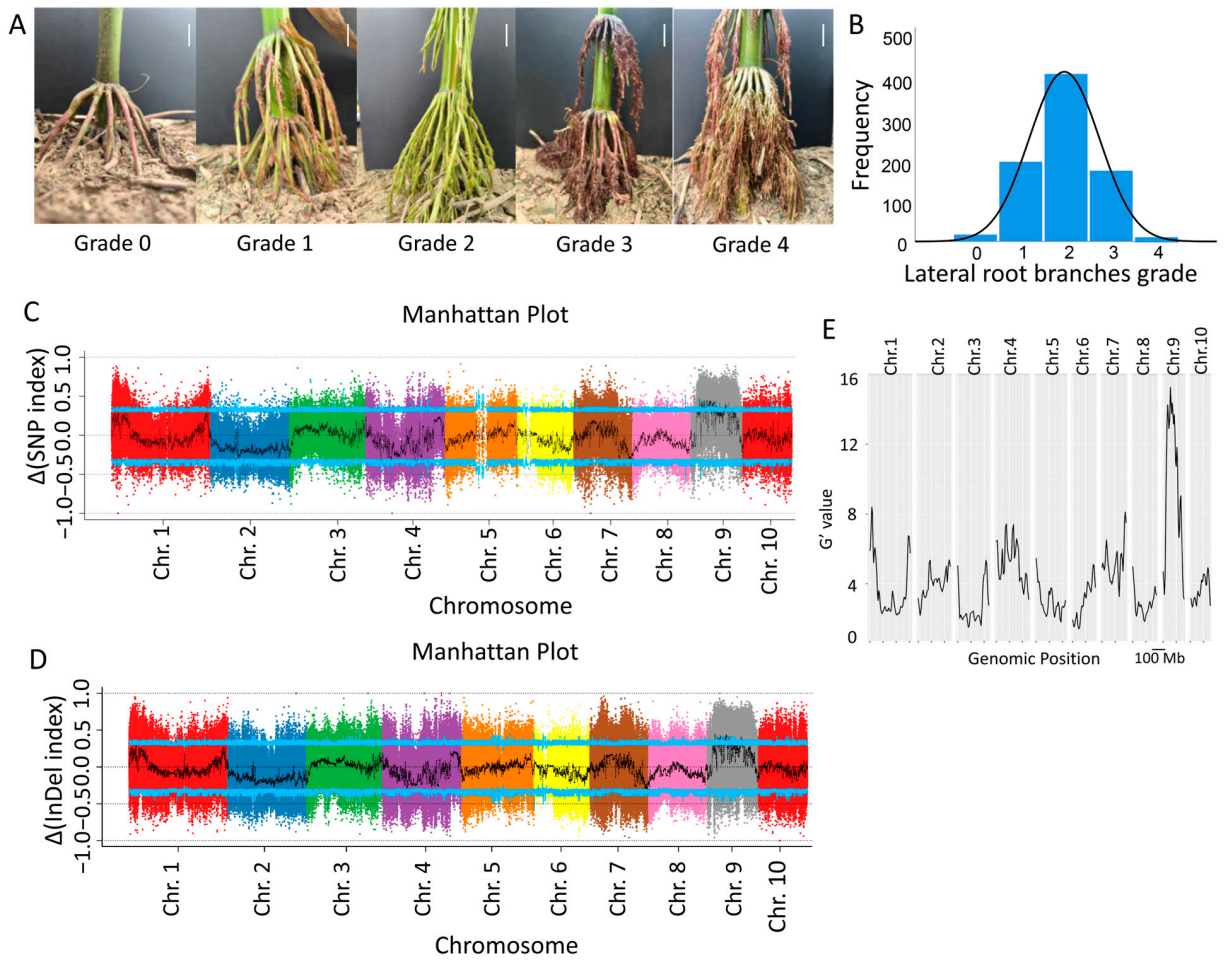
BSA-seq analysis of maize lateral root branches on brace root in the F_2_ population derived from B111 and T4691: (**A**) Phenotypic grading of lateral root branches on brace root in the F_2_ population. (**B**) The distribution of the lateral root branching density in the F_2_ population. (**C**,**D**) ΔSNP-index and ΔInDel-index analysis of maize lateral root branches on brace root based on the BSA-seq data. The blue dot represents the threshold value of significant SNP and InDel at *p* < 0.01 levels, respectively. (**E**) G primer analysis of maize lateral root branches on brace root based on the BSA-seq data.

To identify QTLs associated with lateral root branching, 50 F_2_ individuals with the highest and lowest numbers of lateral roots were selected to construct the high lateral root branching (MLR) and low lateral root branching (LLR) DNA bulks, respectively. The two bulks were subjected to re-sequencing, and the resulting sequence data were analyzed using bulked segregant analysis sequencing (BSA-seq) to map the potential QTL associated with lateral root branching (Figure 3).

The ΔSNP-index and ΔInDel-index were calculated for the two bulks using a sliding-window approach, and their genome-wide distributions were plotted based on the corresponding allele frequencies. A window size of 1 Mb was used to calculate the △SNP-index, followed by 1000 permutation tests to determine the significance threshold. The 99% confidence level (blue line) was used as the screening threshold for QTL detection (Figure 3). Genomic windows exceeding this threshold were significantly associated with lateral root branching. In parallel, genome-wide InDel loci showing significant differences between the two bulks were identified using the criteria of an InDel-index ≥ 0.7 in the MLR bulk (B111-A) and ≤0.3 in the LLR bulk (B111-B).

Based on the combined SNP and InDel analyses, four significant genomic variants were identified, corresponding to two candidate genomic regions (Table 1). Furthermore, G′ (G-prime) analysis indicated that the candidate region on chromosome 9 likely harbors the major-effect QTL controlling lateral root development, which was designated *qLRN9*.

**Figure 4:**
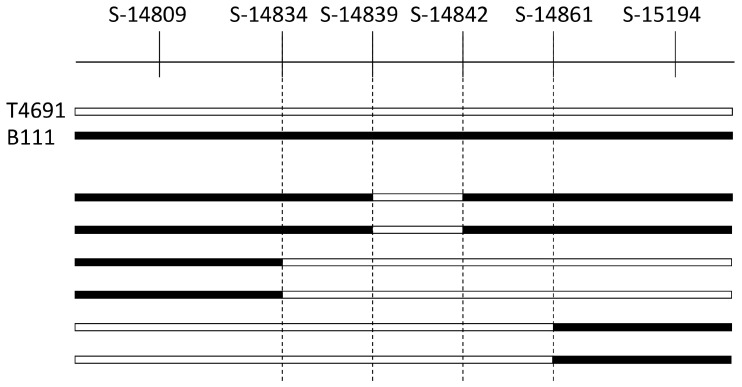
Fine mapping of *qLRN9* by overlapping introgression line mapping.

To further refine the candidate interval of *qLRN9*, 14 InDel polymorphic markers were developed based on sequence differences between the two parental lines and used to genotype the extreme individuals in the F_2_ population (Figure 4; Table S1). By overlapping introgression line mapping, the candidate gene was mapped between markers S-14839 and S-14842 (25,067,662~26,695,367 bp), spanning approximately 1.63 Mb.

### 2.3. Transcriptome Analysis of Maize Lateral Root Development

**Figure 5:**
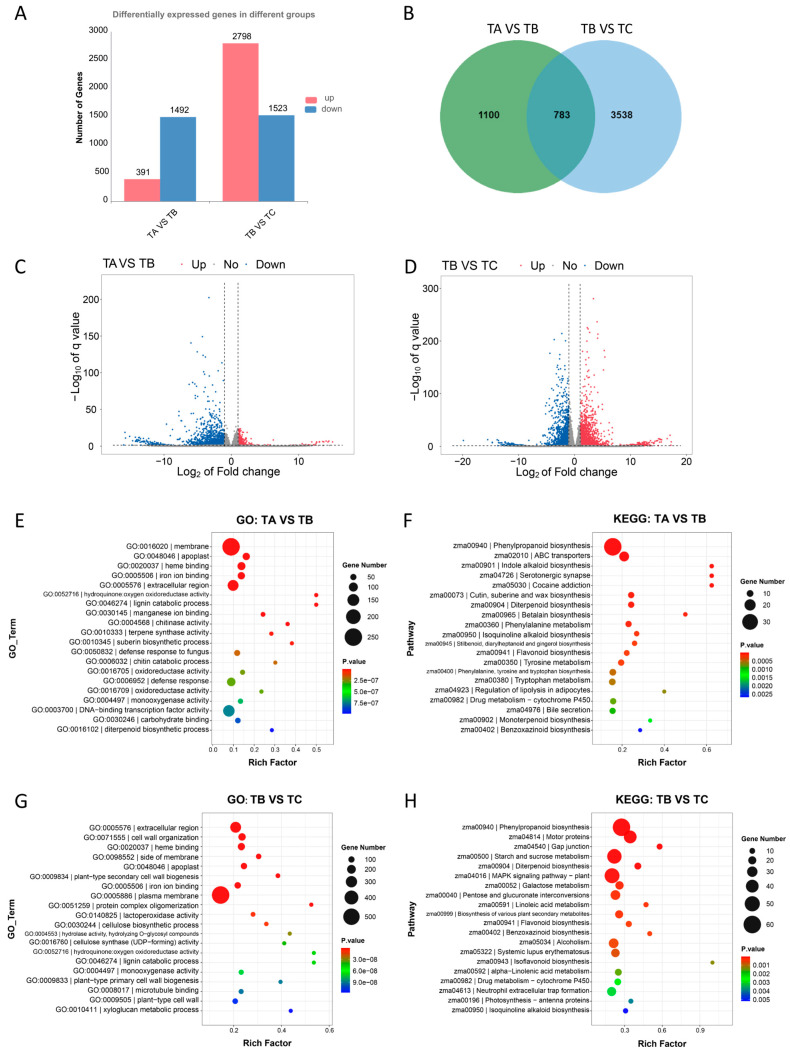
Transcriptome analysis of maize lateral root development in maize inbred line B111: (**A**) DEGs identified in comparative groups TA VS TB and TB VS TC. (**B**) Venn diagram showing the identified DEGs during lateral root development. (**C**,**D**) Heat maps showing gene expression classification of DEGs. Red dots indicate up-regulated genes and green dots indicate down-regulated genes (Significant level: Padj < 0.05, Log2 FoldChange > 1). (**E**,**F**) Gene Ontology (GO) and Kyoto Encyclopedia of Genes and Genomes (KEGG) enrichment analyses of the screened DEGs between TA and TB stages during lateral root development. (**G**,**H**) GO and KEGG enrichment analysis of the screened DEGs between TB and TC stages during lateral root development.

To investigate the transcriptional regulatory mechanisms underlying maize lateral root development, transcriptome analysis was performed at three developmental stages: pre-lateral root primordium formation (TA), lateral root primordium emergence through the epidermis (TB), and lateral root elongation (TC) (Figure 5). A total of 1883 differentially expressed genes (DEGs) were identified between TA and TB stages, including 391 upregulated and 1492 downregulated genes. Gene Ontology (GO) enrichment analysis showed that these DEGs are primarily enriched in terms of membrane, extracellular region, apoplast, iron ion binding, and heme binding. Kyoto Encyclopedia of Genes and Genomes (KEGG) pathway analysis further revealed that DEGs were mainly involved in phenylpropanoid biosynthesis, ABC transporters, cutin, suberine and wax biosynthesis, diterpenoid biosynthesis, and phenylalanine metabolism pathways.

A total of 4321 DEGs were identified between TB stage and TC stages, including 2798 upregulated and 1523 downregulated genes. Between the two comparative groups, there were 783 conserved DEGs. GO enrichment analysis indicated that these DEGs were primarily associated with the plasma membrane, extracellular region, cell wall organization, and heme binding. KEGG pathway analysis showed that DEGs significant enrich in phenylpropanoid biosynthesis, starch and sucrose metabolism, MAPK signaling pathway, and motor proteins.

### 2.4. Integrated Analysis of Gene Mapping and Transcriptome Analysis

**Figure 6:**
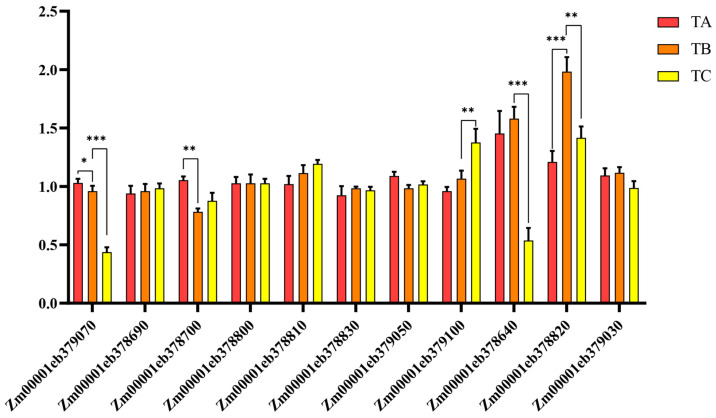
The expression analysis of the putative candidate genes for *qLRN9* during lateral root development by RT-qPCR. Actin was used as the internal control. Error bars represent standard deviation (SD). *, ** and *** indicate that the corresponding gene relative expression levels exhibit significantly different with in TA, TB, TC development stage at *p* < 0.05, *p* < 0.01, *p* < 0.001 levels, respectively.

To identify candidate genes for *qLRN9*, the QTL mapping results were integrated with the transcriptome data. Within the 1.63 Mb candidate interval on chromosome 9 (25,067,662~26,695,367 bp), a total of 43 expressed genes were identified (Table S2). Of these genes, *Zm00001eb378640* (*B-cell lymphoma-2 (Bcl-2) associated athanogene17*, *BAG17*), *Zm00001eb378760*, Zm00001eb378770, *Zm00001eb378820* (*GRF transcription factor13, GRFTF13*), and *Zm00001eb379030* (*kinesin-related protein2*, *KRP2*) were differentially expression between the TB and TC developmental stages (Table 2; Figure 6). GO enrichment analysis of these 43 genes indicated that they were primarily enriched in terms of ATP binding, protein transport, plasma membrane, and metal ion binding.

Based on gene functional annotations, tissue-specific expression patterns, and transcript abundance, eight candidate genes were screened out from the 43 expressed genes within the fine-mapping interval (Table 2; Figure 6). Of them, *Zm00001eb379070*, encoding cellulose synthase-like protein D2, was highly expressed at stage TA and was gradually downregulated during lateral root development. *Zm00001eb379050*, encoding *defender against cell death 1* (*DAD1*), maintained relatively high expression levels throughout lateral root development. *Zm00001eb378690* (*Probable transcriptional regulator SLK2-like*), *Zm00001eb379100* (encoding ACD11-binding partner), *Zm00001eb378700* (*RING-type E3 ligase509, RING509*), *Zm00001eb378800* (*multiple organellar RNA editing factor5*, *MORF5*) and *Zm00001eb378830* (encoding endoplasmic reticulum metallopeptidase) exhibited relatively stable expression patterns across the three developmental stages. In contrast, the expression of *Zm00001eb378810*, encoding Elongator complex protein 6, which is involved in polar auxin transport, increased progressively during lateral root development. To validate the transcriptome data, 11 representative genes, including the eight candidate genes, were selected for RT-qPCR analysis. The RT-qPCR results were largely consistent with the transcriptome data, confirming the reliability of the RNA-seq expression profiles.

To further identify the candidate genes for *qLRN9*, six genes were selected for gene sequence alignment analysis between the parental inbred lines B111 and T4691 (Table 2). Sequence comparison revealed polymorphisms in four of the six genes examined. Specifically, *Zm00001eb378700*, *Zm00001eb378820*, and *Zm00001eb379070* each contained SNPs between the two tested inbred lines, whereas *Zm00001eb379100* harbored six SNPs and a 27 bp insertion in B111 relative to T4691. Moreover, these four genes also exhibited differential expression in lateral root development, among which are the possible candidate genes for *qLRN9*. For *Zm00001eb379100* harbor a 27 bp insertion, it is probably the most promising candidate gene.

## 3. Discussion

Lateral roots are essential components of the plant root systems. In monocots, lateral roots constitute a major proportion of the fibrous root system, and therefore largely determine the functions of the root system [23]. In maize, post-embryonic root development begins with the formation of the primary root and seminal roots, followed by the development of adventitious crown roots, brace roots, and lateral roots [24]. In the present study, we identified the maize inbred line B111, which exhibits abundant lateral roots on both the underground root system and brace roots. This unique phenotype suggests that B111 may harbor favorable genetic variants associated with lateral root initiation and development, especially the lateral root formation on brace roots. Therefore, the inbred line B111 is a valuable genetic resource for dissecting the regulatory mechanisms underlying maize lateral root development. The length and branching density of maize lateral roots are important determinants of drought adaptation [17,25]. Previous studies have demonstrated that appropriate modifications of lateral root architecture, including reduced lateral root branching density and increased lateral root length, can enhance drought resilience by improving soil exploration and water acquisition. In this study, F_2:3_ lines with high lateral root branching density exhibited enhanced drought tolerance and significant influence on root development. Whereas lines with sparse lateral roots showed more pronounced effects on shoot growth. These results indicate that increased lateral root branching density contributes to improved drought tolerance in maize, potentially by maintaining root system functionality under water-limited conditions.

To date, researchers have performed a lot of genetic analyses to identify loci or genes for maize root development [26,27,28,29,30,31]. In the primary mapping chromosomal region of *qLRN9*, QTLs or significant SNPs have been identified for primary root weight [32], lateral root length [32], crown/brace root number [29,33,34], and total root length [35]. And, several genes have been functional identified for controlling lateral root development, such as *ZmbZIP4* [36], *defective in lateral root 2* (*ZmDLR1*) [37], *ZmARF1* [38], *ZmLRT* [39], *LATERAL ROOT PRIMORDIA 1* (*LRP1*) [40], *lateral rootless 1* (*lrt1*) [41], *ZmSULTR3;4* [42], *ZmNAC1* [43], *haem oxygenase-1 (ZmHO-1)* [44], *ZmARF4* [45], and *short lateral roots1 and 2 (slr1 and slr2)* [46]. However, there is not any lateral root development-related gene has been identified in the fine mapping interval of *qLRN9*. These results revealed that the chromosomal region of *qLRN9* may harbor putative regulatory genes for lateral root development.

Increasing research indicates that lateral root development is regulated by complex interactions among endogenous and environmental factors, including phytohormones, nutrient status, drought stress, light, gravity and mechanical signals [23,47,48,49]. Only a limited number of pericycle cells acquire lateral root founder cell identity and initiate lateral root formation [50]. Among the regulatory pathways involved, the auxin signaling pathway plays a central role in lateral root initiation [51,52]. Reactive oxygen species (ROS) have been proposed to function as important signaling during auxin-regulated lateral root development [53,54,55]. In *Arabidopsis*, ACCELERATED CELL DEATH 11 (ACD11) and its interacting partners participate in programmed cell death and ROS-mediated defense response [56,57]. However, the biological functions of ACD11-related proteins in maize remain largely unknown. In this study, an ACD11-binding partner encoding gene, *Zm00001eb379100*, was identified within the *qLRN9* interval. Notably, sequence comparison revealed a 27 bp insertion in B111 compared with T4691, suggesting that allelic variation in this gene may contribute to differences in lateral root development.

In plants, RING-type E3 ligases play central roles in response to different intrinsic and environmental signals [58], are involved in the regulation of flowering time [59,60], leaf development [58], phosphate homeostasis and root development [61,62,63,64], multiple stress responses [65,66,67,68], and abscisic acid signaling [69,70]. However, the functions of RING-type E3 ligases in maize are still unclear. In this study, a RING-type E3 ligase encoding gene, *Zm00001eb378700*, was identified in the fine mapping interval of *qLRN9*. These results revealed that *Zm00001eb378700* likely plays an important role in lateral root development.

Cell wall remodeling is another essential process involved in multiple stages of lateral root development, including lateral root founder cell identity, initiation, growth, and emergence [71]. These developmental processes require coordinated regulation of cell wall synthesis, degradation, and rearrangement [72]. Within the fine-mapping interval, *Zm00001eb379070*, encoding cellulose synthase-like protein D2, exhibited high expression levels during lateral root development, suggesting that it may participate in regulating cell wall-associated processes during lateral root formation. Growth-regulating factors (GRFs) are important transcriptional regulators involved in diverse aspects of plant growth and development, including shoot and root formation [73,74]. The conserved miR396-*GRFs* regulatory module has been shown to regulate root regeneration and development in multiple plant species [75,76,77]. In the present study, *GRFTF13* (*Zm00001eb378820*) exhibited differentially expression in lateral root development, indicating that it may represent another potential regulator contributing to the *qLRN9*-associated phenotype.

Collectively, our integrated QTL mapping and transcriptome analyses identified *Zm00001eb379100*, *Zm00001eb378700*, *Zm00001eb379070*, and *GRFTF13* as promising candidate genes underlying *qLRN9*. These genes are involved in biological processes closely associated with root development, including auxin transport, ROS signaling, cell wall regulation, and transcriptional control. However, further genetic and functional validation, including gene editing and complementation analysis, is required to determine the causal gene responsible for variation in maize lateral root branching density.

In conclusion, our study demonstrates that increased lateral root branching density enhances maize drought tolerance. Using BSA-seq combined with molecular marker analysis, we identified a major-effect QTL for maize lateral root branching density and narrowed it to a 1.63 Mb marker interval. Integration of QTL mapping and transcriptome analyses further identified four putative candidate genes within the region, and *Zm00001eb379100* is probably the most promising candidate gene. However, the final candidate gene still needs to be identified and functionally validated. These findings provide new insights into the genetic regulation of maize root architecture and establish a foundation for future molecular breeding strategies aimed at improving drought resilience.

## 4. Materials and Methods

### 4.1. Plant Materials and Phenotyping

The maize inbred lines B111 and T4691 were used in the present study. The inbred line B111, which exhibits dense lateral root branching, was selected from an association mapping population [78]. The inbred line T4691 is an elite maize inbred line that was bred by our group. To identify QTLs for maize lateral root branching, an F_2_ population was generated by crossing B111 and T4691. In 2024, a total of 812 F_2_ individuals were grown in the field for phenotypic evaluation, BSA-seq and self-pollination.

Because accurately quantifying underground lateral roots is technically challenging, brace roots were used as a convenient proxy for evaluating lateral root branching in this study. Both B111 and some F_2_ individuals exhibited abundant lateral root branches on brace roots, facilitating phenotypic assessment. Phenotyping and sample collection were performed at 20 days after pollination, when brace root growth and lateral root development had largely ceased. Based on lateral root branching density on brace roots, F_2_ individuals were classified into five phenotypic categories (Grades 0–4).

The grading criteria and corresponding phenotypic characteristics were defined as follows: Grade 0, smooth brace root surface without visible lateral root primordium protrusions.; Grade 1, emergence of a small number of lateral root primordia, representing the initial stage of lateral root formation; Grade 2, increased lateral root primordia formation, with some lateral roots penetrating the epidermis and undergoing limited elongation; Grade 3, substantially increased lateral root number accompanied by obvious elongation; Grade 4, dense and fully elongated lateral roots or lateral root branches, representing the highest level of lateral root branching.

### 4.2. Investigation of Drought Tolerance at the Seedling Stage

To evaluate the effects of lateral root branching density on drought tolerance, eight F_2_ individuals exhibiting either high or low lateral root branching density were selected for self-pollination to generate F_2:3_ lines. The resulting F_2:3_ lines with relatively high lateral root branching (MLR) and low lateral root branching (LLR) were subjected to drought stress treatment. For every F_2:3_ line, 100 seeds were surface sterilized with 1% sodium hypochlorite solution for 10 min, rinsed thoroughly with sterile water, and germinated in darkness at 30 °C. Two-week-old pot-grown maize seedlings were then subjected to water-limitation treatments with twenty plants each for three replicates, following a previously described method [79]. After drought treatment, all the seedlings were collected for measuring average root diameter and lateral root branching using an Epson Expression 12000XL root scanner (Seiko Epson Corporation, Suwa, Japan) coupled with WinRHIZO Pro software [80]. Above-ground and underground tissues were separately collected, oven-dried, and weighed to determine biomass allocation and calculate the root-to-shoot ratio.

### 4.3. DNA Extraction, BSA-Seq and Molecular Marker Analysis

Based on the lateral root branching phenotypes, all 812 individuals in the F_2_ population were evaluated. Fifty F_2_ individuals exhibiting the highest and lowest lateral root numbers were selected to construct two extreme bulks, designated as the MLR and LLR bulks, respectively. Fresh leaf samples of the two extreme bulks and two parental lines were collected for genomic DNA extraction using the SLS method [81]. The obtained DNA samples were subjected to BSA-seq analysis using the Illumina HiSeq2500 platform (Berry Genomics, Beijing, China). The original sequencing data in fastq format have been deposited in the Genome Sequence Archive (GSA) database of the National Genomics Data Center (accession number: CRA044543, https://ngdc.cncb.ac.cn/gsa/s/r0Rz2cUW, accessed on 10 June 2026).

High sequencing quality was obtained, with Q20 and Q30 values exceeding 97.42% and 92.84%, respectively. The GC content ranged from 46.23% to 46.56%, indicating qualified library construction and a balanced nucleotide composition. Clean reads were aligned to the maize B73 RefGen_v5 using the BWA software package (bwa-0.7.19). SNP and InDel variants were identified using GATK [82], and annotation was performed using ANNOVAR to determine their genomic locations [83]. A total of 29,428,171 SNPs and 1,597,786 InDels were identified. Homozygous polymorphic markers between the two parental lines were screened from genotyping results. To reduce potential errors caused by sequencing and read alignment, SNP loci with progeny SNP-index values below 0.3, sequencing depth less than 7×, or missing SNP-index values were removed. To improve mapping resolution, reduce background noise from individual SNPs, and genomic regions associated with lateral root branching, the ΔSNP-index and ΔInDel-index values were calculated using a sliding-window-based Gprime analysis [84]. Gprime values were calculated using a 1 Mb sliding window and 10 kb step size.

### 4.4. Total RNA Extraction, Transcriptome Sequencing Analysis, qRT-PCR

The maize inbred line B111 was used for transcriptome sequencing analysis. Based on the development progression of lateral roots, samples were collected at three consecutive stages with three biological replicates: before lateral root primordium initiation (stage TA), lateral root primordium emergence (stage TB), and lateral root elongation (stage TC). All samples were immediately frozen in dry ice and subsequently stored at −80 °C until RNA extraction. For transcriptomic sequencing, total RNA of all the lateral root samples was extracted using Trizol reagent (Invitrogen, Waltham, MA, USA) according to the manufacturer’s instructions. RNA sequencing libraries were constructed and sequenced on an Illumina HiSeq 4000 (LC-Bio Technologies, Hangzhou, China) following the standard Illumina protocols [85,86]. The raw transcriptome sequencing data in FASTQ format have been deposited in the Genome Sequence Archive (GSA database) National Genomics Data Center under accession number CRA044543 (https://ngdc.cncb.ac.cn/gsa/s/r0Rz2cUW, accessed on 10 June 2026).

Clean reads were aligned to the maize reference genome using HISAT2 (https://daehwankimlab.github.io/hisat2/, version: hisat2-2.2.1, accessed on 10 June 2026). Transcript assembly and quantification were performed using StringTie (http://ccb.jhu.edu/software/stringtie/, version: stringtie-2.1.6, accessed on 10 June 2026) with default parameters. Gene expression levels were normalized to fragments per kilobase of transcript per million mapped reads (FPKM). Differentially expressed genes were identified using the DESeq2 R package (v1.40.2) and edgeR (v3.42.4). Genes with a false discovery rate (q-value) < 0.05 and fold change (FC) ≥ 2 were considered significantly upregulated, whereas genes with q-value < 0.05 and FC < 0.5 were considered significantly downregulated. Gene Ontology (GO) and Kyoto Encyclopedia of Genes and Genomes (KEGG) enrichment analyses were conducted using clusterProfiler (v4.2.2). The top 20 significantly enriched pathways were selected based on *p*-values and visualized using bubble plots generated with ggplot2 (v3.5.1) software at the OmicStudio cloud platform provided by LC-Bio Technologies. Statistical tests were performed using default two-sided settings, and enrichment significance was evaluated using default parameters.

For RT-qPCR validation, total RNA of the samples collected at three consecutive stages was treated with DNase I to remove genomic DNA contamination, followed by first-strand cDNA synthesis using RevertAid reverse transcriptase (Thermo Fisher Scientific, Waltham, MA, USA) with oligo(dT) primers. Quantitative PCR was performed using SYBR Premix ExTaq II kit (RR820A, TAKARA, San Jose, CA, USA) on a CFX96 Real-Time PCR Detection System (BioRad, Hercules, CA, USA) for three technical replicates. The primers used in this study are listed in Table S3.

### 4.5. Statistical Analyses

All the collected data from phenotypic analysis and qRT-PCR analysis were subjected to one-way variance analysis (ANOVA) and Student’s *t*-test using software SPSS 22.0 (IBM, Armonk, NY, USA). *p* < 0.05 indicates the statistical differences reach the significantly different level; *p* < 0.01 indicates very significantly different levels.

## Figures and Tables

**Table 1:** QTLs for maize lateral root branches mapped by BSA-seq in the F_2_ population derived from B111 and T4691.

QTL	Maker Type	Chromosome	Start	End	Region Size (Mb)
*qLRN7*	ΔSNP-index	7	16,998,001	17,746,600	0.75
	ΔIn Del-index	7	169,463,001	178,018,000	8.55
*qLRN9*	ΔSNP-index	9	16,242,001	114,729,000	98.49
	ΔIn Del-index	9	16,250,001	133,881,000	117.63

**Table 2:** The possible candidate genes screened in the fine mapping interval of *qLRN9*.

Gene ID	Description	Main GO Terms	Differential Expression in Transcriptome or RT-qPCR Analysis	Variants in B111
*Zm00001eb378640*	protein binding protein *BAG17*	Protein-folding chaperone binding; Cytoplasm protein quality control by the ubiquitin-proteasome system	Yes	Undetected
*Zm00001eb378760*	uncharacterized protein LOC100278437	NA	Yes	Undetected
*Zm00001eb378770*	probable receptor-like protein kinase At2g21480 precursor	NA	Yes	Undetected
*Zm00001eb378820*	GRF-transcription factor 13	ATP binding; Developmental process; DNA-templated transcription	Yes	7 SNPs
*Zm00001eb379030*	kinesin-related protein2	Microtubule; Microtubule motor activity; Cell division	No	Not detected
*Zm00001eb378690*	Probable transcriptional regulator SLK2-like	Cell differentiation; Gynoecium development; regulation of response to osmotic stress	No	Undetected
*Zm00001eb378700*	RING-type E3 ligase509	Metal ion binding; Ubiquitin-conjugating enzyme binding; Negative regulation of cell population proliferation	Yes	7 SNPs; A 3 bp insertion; A 3 bp deletion
*Zm00001eb378800*	DAG protein	Chloroplast; Protein dimerization activity	No	Undetected
*Zm00001eb378810*	Elongator complex protein 6	Elongator holoenzyme complex; Auxin polar transport; Root morphogenesis	No	Undetected
*Zm00001eb378830*	endoplasmic reticulum metallopeptidase 1	Endoplasmic reticulum membrane; Metal ion binding	No	Undetected
*Zm00001eb379050*	false DAD1	Oligosaccharyltransferase complex; Apoptotic process	No	Undetected
*Zm00001eb379070*	Cellulose synthase-like protein D2	Golgi membrane; Cellulose synthase (UDP-forming) activity; Cell wall organization	Yes	7 SNPs
*Zm00001eb379100*	Binding partner of ACD11 1	Plasma membrane; Defense response to fungus; Negative regulation of plant-type hypersensitive response	Yes	6 SNPs; A 27 bp insertion

Note: NA indicates that the gene was not enriched in any GO term.

## Data Availability

The original BSA-seq and transcriptomic sequencing data in fastq format have been uploaded to the National Genomics Data Center GSA database (accession number: CRA044543, https://ngdc.cncb.ac.cn/gsa/s/r0Rz2cUW, accessed on 10 June 2026).

## Supplementary Materials

The following supporting information can be downloaded at: https://www.mdpi.com/article/10.3390/ijms27156962/s1.
